# Complementary and Alternative Medicine Use Among Families of Children with Autism Spectrum Disorder Attending Five Autism Centres in Arab Countries: Associations with Sociodemographic Factors and Health Literacy

**DOI:** 10.3390/healthcare14193351

**Published:** 2026-10-08

**Authors:** Mais Hatahet, Nguyen Minh Chau, Andrea Sárváry, Attila Sárváry

**Affiliations:** 1 Doctoral School of Health Sciences, University of Debrecen, 4032 Debrecen, Hungary; mhatahet@hamad.qa; 2 Department of Health Informatics, Institute of Health Sciences, Faculty of Health Sciences, University of Debrecen, 4032 Debrecen, Hungary; nguyem.chauminh@etk.unideb.hu; 3 Department of Psychology, Institute of Social Sciences, Faculty of Health Sciences, University of Debrecen, 4400 Nyíregyháza, Hungary; sarvary.andrea@etk.unideb.hu; 4 Department of Nursing and Integrative Health Sciences, Institute of Health Sciences, Faculty of Health Sciences, University of Debrecen, 4400 Nyíregyháza, Hungary

**Keywords:** autism spectrum disorder, complementary and alternative medicine, children, health literacy

## Abstract

**Background:** Complementary and alternative medicine (CAM) therapies are commonly used among children with autism spectrum disorder (ASD). This study aimed to examine parental attitudes toward CAM, in addition to patterns of its use and associated factors among children with ASD in Arab countries. **Methods:** In this cross-sectional survey, 142 questionnaires from five Arab countries (Qatar, the Kingdom of Saudi Arabia [KSA], Kuwait, Jordan, and Libya) were analysed. **Results:** The most reported CAM modalities included dietary supplements such as multivitamins and fish oil, as well as the questionnaire item labelled “holding therapy”. The main reason for CAM use reported by parents was the desire to try all possible therapy options for their child and reduce stress (35.5%), followed by improving their child’s quality of life (32.6%). The regression models showed that better verbal ability was associated with greater use of these therapies (use of short/complex sentences vs. does not talk: biologically based therapies, OR = 5.35, 95% CI 1.58–18.56; mind and body practices, OR = 4.30, 95% CI 1.66–11.23). The models also showed an association between the male sex and the use of mind and body practices (OR = 3.28, 95% CI 1.23–8.92). **Conclusions:** CAM use was common in this sample. Healthcare professionals should provide balanced, evidence-based information and encourage non-judgmental discussion of CAM use. Improving health literacy and access to reliable information may support safer decision-making.

## 1. Introduction

Autism spectrum disorder (ASD) is a neurodevelopmental condition characterised by differences in social communication and interaction, together with restricted or repetitive patterns of behaviour, interests, or activities [1].

The reported prevalence of ASD has increased substantially in recent decades, as documented by population-based surveillance systems such as the CDC Autism and Developmental Disabilities Monitoring (ADDM) Network [2,3]. Prevalence rates vary across countries and studies, which can be attributed to differences in diagnostic practices, surveillance systems, study populations, and methodology. Evidence from Arab countries remains comparatively limited and heterogeneous, highlighting the need for further research in these settings.

Complementary and alternative medicine (CAM) is heavily used worldwide, with estimates of CAM use in the general population ranging from 10% to 76% [4,5]. In the context of ASD, parents and families may seek CAM as an alternative or complementary option; for example, in an effort to avoid medications and their potential adverse effects [6,7].

A recent study in Turkey reported that more than 50% of parents of children with ASD were using or had used at least one CAM practice, either as a substitute for or alongside conventional medicine [5]. Diets, dietary supplements, vitamins, and probiotics were among the most used CAM approaches. Some parents reported the belief that even if these approaches provided no benefit, they were unlikely to cause harm [8]. However, the growing emphasis on evidence-based practices (EBPs) reflects the need for rigorous research on interventions for autism. At the same time, interventions with limited evidence of efficacy may continue to be described as “innovative” highlighting an important gap in the current knowledge [9].

Limited data are available on parental attitudes toward CAM and the prevalence of its use among children with ASD in Arab countries. A study conducted in Jordan found that 47% of parents used CAM modalities for their children with ASD, and parents with higher levels of education were more likely to access rehabilitation services [10]. In another cross-sectional study conducted in Saudi Arabia, parental education level and receipt of more hours of conventional services or access to rehabilitation centres were associated with increased CAM use among children with ASD [11].

This study aimed to examine parents’ use of CAM for children with ASD attending five participating autism centres, including one each in Jordan, Qatar, the Kingdom of Saudi Arabia, Kuwait, and Libya. We also investigated associations between CAM use and child-related factors (age, sex, medical variables, and verbal ability), parental sociodemographic factors, and health literacy. The study was designed as a centre-based multicountry survey using the same questionnaire and analytical approach across the participating settings; it was not intended to provide nationally representative prevalence estimates.

## 2. Materials and Methods

### 2.1. Sample and Sampling Method

**Figure 1:**
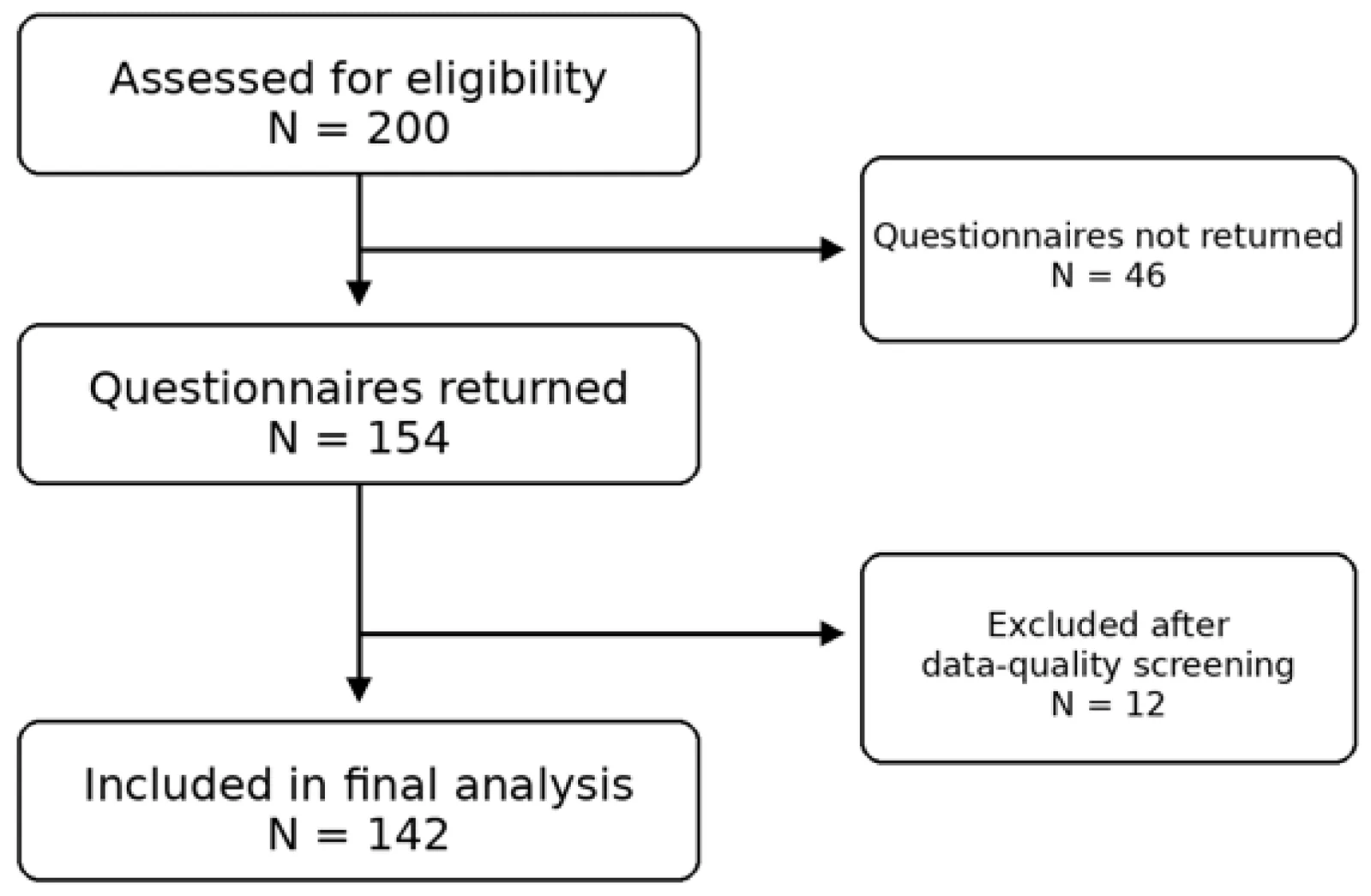
Flowchart of participant selection.

We used convenience sampling in this cross-sectional study. The Autism Academy of Jordan, in collaboration with the Jordanian Association for Autism, facilitated contact with participating autism centres in the other countries. Five centres participated, including one each in Jordan, Qatar, the Kingdom of Saudi Arabia, Kuwait, and Libya. The online questionnaire was distributed through the participating centres, beginning in Jordan and subsequently extending to the other participating centres as recruitment progressed (200 parents were assessed for eligibility). A total of 154 questionnaires were returned. Following data quality screening, 12 questionnaires were excluded because of incomplete responses, duplicate submissions, or logical inconsistencies, leaving 142 completed questionnaires for the final analysis (Figure 1). The analytical dataset did not retain a reliable centre/country identifier for individual respondents; therefore, the exact final distribution of the 142 participants across the five participating centres could not be reconstructed retrospectively.

Screening was performed to identify duplicate submissions, incomplete responses, and logical inconsistencies.

### 2.2. Survey Distribution

Responsible personnel at the participating centres assisted with survey distribution between January and July 2022. Occupational therapy practitioners at the centres identified potentially eligible families according to the study criteria. Parents received information about the study and a link to the self-administered Google Forms questionnaire. Mothers were asked to complete the questionnaire on behalf of the family. Eligible participants were parents of a child who had been diagnosed with ASD at least 6 months before the study. Questionnaires with incomplete information or responses that did not meet the inclusion criteria were excluded.

### 2.3. Study Questionnaire

A cross-sectional survey was distributed using a self-administered Google Forms questionnaire. The study-specific CAM questionnaire was prepared in English, translated into Arabic by an expert, and reviewed by two experts in the field of ASD. Health literacy was assessed separately using the Arabic version of the HLS-EU-Q16. The introductory section provided detailed information about the study aims, voluntary participation, consent, and contact details.

The questionnaire included the following sections:

1. Demographic data of the parents (age, number of children, family income, educational level, and place of residence) and their child with autism (age, sex, and age at diagnosis).
2. Clinical characteristics of the child with autism, including:
  - The exact diagnosis (reported by the parents);
  - The comorbid syndromes of the child with ASD;
  - The child’s verbal ability level.
3. CAM use by parents for their child with ASD. The questionnaire included CAM modalities reported in the previous literature on ASD/CAM and organised using the operational taxonomy applied in this study, informed by the National Center for Complementary and Integrative Health (NCCIH) framework and previous studies [10,12,13,14,15,16,17,18,19]. Respondents selected the CAM practices used for their child. For the regression analyses, modalities were grouped into two broad study categories: (a) natural products/biologically based therapies and (b) mind and body practices. Because CAM taxonomies have evolved and some modalities can be classified differently across sources or may also be used in conventional rehabilitation/supportive care, these categories should be regarded as operational study groupings rather than a universal classification. Definitions, study categories, and supporting sources for all included modalities are provided in Supplementary Table S1.
4. Information sources regarding CAM (categories: social media, friends, families who have children with autism, doctor, rehabilitation therapists, TV/radio, and others).
5. Reasons for CAM use.
6. Disclosure of CAM use to the physician. Participants were asked to indicate whether they had informed the physician about their child’s CAM use, and if not, to state the reason.
7. Health literacy, which was assessed using the Arabic version of the HLS-EU-Q16 [20,21]. In the present survey, five response categories were used: “very difficult”, “fairly difficult”, “neutral”, “fairly easy”, and “very easy”. For analysis, “very difficult” and “fairly difficult” were coded as 0, whereas “fairly easy” and “very easy” were coded as 1, producing a total score ranging from 0 to 16, while “neutral” was omitted from the analyses. Scores were categorised descriptively as inadequate (0–8), problematic (9–12), and sufficient (13–16). However, because inclusion of a “neutral” response option represents a modification of the standard HLS-EU-Q16 response format, these categories should not be considered strictly equivalent to classifications derived using the standard response format. The present sample had good internal consistency (Cronbach’s α = 0.84) [20,21].

### 2.4. Validity and Reliability of the Instrument

Two academic experts in the field of ASD and 10 parents were asked to complete and review the questionnaire to determine whether the questions were appropriate, comprehensible, and understandable before distribution.

### 2.5. Statistical Methods

We use descriptive statistics to present the baseline characteristics of the sample and the prevalence of CAM use. Multiple binary logistic regression models were used to examine the associations between CAM use and child sex, parental education level, comorbidity, medication use, child verbal ability, and health literacy. Explanatory variables were selected based on the literature. The sample size at the country level was too small to include country/site reliably in the models. As medication use and side effects showed collinearity, only the former was retained. Household income was not included because 21.1% of respondents selected “Prefer not to answer” Penalised logistic regression was used to calculate adjusted odds ratios (ORs) with 95% confidence intervals using the “brglm” package in R statistical software (version 4.4.1). Analyses were performed using SPSS (version 27.0, IBM Corp., Armonk, NY, USA), Intercooled Stata v17 (StataCorp, College Station, TX, USA, 2021), and R (version 4.4.1). Model selection using the Akaike information criterion (AIC) was performed to identify the best-fitting model, with lower AIC values indicating better balance between model fit and complexity [22]. The significance threshold was set at *p* < 0.05.

### 2.6. Ethical Approval

Prior to data collection, the study protocol and ethical documentation were internally reviewed and approved by the Research and Development Committee of the Autism Academy of Jordan (AAJ), an internal research review body rather than a formal institutional review board/research ethics committee. Organisational authorisation to distribute the questionnaire and conduct the study was also obtained from the participating organisations/centres.

Ethical oversight and authorisation for the study were documented through the participating organisations before data collection. The manuscript records approval/authorisation from the Jordanian Association for Autism (13 December 2021), the Department of Occupational Therapy at Hamad Medical Hospital in Qatar (9 January 2022), and the Autism Academy of Jordan (Ref. 72/2022; 25 January 2022), which coordinated with partner centres in the Kingdom of Saudi Arabia (Stepping Stones, Riyadh), Kuwait (First Steps Center), and Libya (National Centre for Diagnosis and Treatment of Autism). Participation was voluntary, and electronic informed consent was obtained from participants before completion of the questionnaire. Data were collected anonymously; no personally identifiable information was obtained. Study data were treated confidentially, stored securely, and used solely for research purposes.

## 3. Results

### 3.1. Sociodemographic Characteristics of the Parents

A total of 142 parents of children with ASD participated in the survey. The mean age of the fathers was 44.5 years (range: 23–67 years), and the mean age of the mothers was 38.8 years (range: 23–66 years). More than 60% of parents had a higher education level (Table 1).

### 3.2. Sociodemographic Characteristics of the Children with ASD

Most children were male. The mean age of the children was 8.4 years (range: 1–17 years), with approximately 70% aged 5–14 years and nearly one quarter aged 1–4 years (Table 1). The mean age at diagnosis was 2.5 years (SD 1.1), and the mean number of siblings was 3 (SD 1.27).

### 3.3. Clinical Characteristics of Children with ASD

Most children were diagnosed with autism disorder (125; 88.0%), while the remaining children were reported as having pathological demand disorder, Asperger syndrome, or another diagnosis. More than 40% were unable to talk. The most frequently reported comorbidity was attention-deficit/hyperactivity disorder (ADHD) (77.5%) (Table 2).

### 3.4. Parents’ Health Literacy

Health literacy was classified as inadequate in 44.4% of parents, problematic in 27.5%, and sufficient in 28.1%.

### 3.5. CAM Use Among Children with ASD

A total of 88.7% of parents reported using some form of CAM for their children. Natural products/biologically based therapies had been used by 104 respondents (73.2%), while 100 (70.4%) reported using mind and body practices. The most reported individual modalities were multivitamins (75; 52.8%) and the questionnaire item labelled “holding therapy” (73; 51.4%). About 40% of respondents used fish oil and facilitated communication, and 30% applied honey, pet therapy, vitamin B6, dates, and sensory integration therapy in the ever-used group (Table 3). Among the dietary approaches, gluten-free and low-sugar diets were the most commonly reported.

During the previous 6 months, the most frequently reported modalities were facilitated communication (17.6%), fish oil (12.7%), and the questionnaire item labelled “holding therapy” (10.6%).

Among the parent-reported perceived effectiveness ratings shown in Table 3, the questionnaire items labelled “holding therapy” (45.2%) and “facilitated communication” (38.2%) had the highest reported proportions of positive ratings. These subjective ratings only describe parental perceptions and should not be interpreted as evidence of clinical efficacy. For facilitated communication, the proportions rated ineffective and effective were the same.

Parents were also asked about their reasons for using CAM for their children with ASD. The most reported reason was the desire to try everything possible for their child and reduce stress (49; 35.5%), followed by improving the child’s quality of life (45; 32.6%). Approximately one third of parents (40; 29.0%) selected “positive experiences recommended by many families”. More than one fifth reported using CAM to reduce medication side effects (32; 23.2%) or because they were uncertain whether conventional therapy alone could help (33; 23.9%).

### 3.6. Disclosure of CAM Use

Most parents (67.6%) reported disclosing all or some CAM methods used for their child. Among the reasons for non-disclosure, 31.7% reported that physicians had never asked about CAM use, while 28.2% believed that physicians had little knowledge about CAM.

### 3.7. Information Sources of CAM

Almost 60% of parents obtained information about CAM through social media (59.2%), while 30.3% obtained information from families of children with autism. Other sources included rehabilitation therapists (20.4%), friends (18.3%), physicians (16.9%), and TV/radio (7.7%).

### 3.8. Associations Between Sociodemographic and Medical Factors, Health Literacy, and CAM Use

The results of the best-fitting binary logistic regression models are presented in Table 4. No clear associations were observed between child age group or parental education and the main CAM groups in this exploratory model. The model showed an association between male sex and the use of mind and body practices (OR = 3.28, 95% CI 1.23–8.92).

Better verbal ability was also associated with greater use of both main CAM groups (short/complex sentences vs. does not talk: OR = 5.35, 95% CI 1.58–18.56 for natural products/biologically based therapies; OR = 4.30, 95% CI 1.66–11.23 for mind and body practices). The wide confidence intervals indicate substantial uncertainty in these estimates.

A sensitivity analysis was conducted to assess the influence of potentially ambiguous or controversial questionnaire items on the mind and body practices model. The primary model included all modalities listed in Table 3, as presented in Table 4. In the sensitivity analysis, holding therapy, facilitated communication, and packing therapy were excluded from the mind and body practices category. The resulting model showed statistically significant associations between CAM use and medication use (OR = 2.53, 95% CI 1.00–6.38; *p* = 0.048) and between CAM use and the child’s verbal ability (short/complex sentences vs. does not talk: OR = 6.55, 95% CI 1.65–25.95; *p* = 0.007).

## 4. Discussion

In this study, we investigated parents of children with ASD to explore their attitudes toward CAM use and associations between selected factors and CAM use across five centres in five Arab countries. Approximately 90% of parents reported using at least one CAM modality for their child. Commonly reported approaches included use of dietary supplements such as multivitamins, fish oil and the questionnaire item labelled “holding therapy” The questionnaire did not provide a sufficiently detailed operational description of “holding therapy”; therefore, the high reported frequency cannot be assumed to represent a single standardised intervention. In the literature, coercive holding or attachment-based therapies may involve physical restraint and have raised serious safety and ethical concerns; these practices should not be conflated with supportive holding or non-coercive deep-pressure sensory input [23,24]. The available evidence is insufficient to establish clinical efficacy for coercive holding approaches, and reports of adverse events reinforce the need for caution [23,24].

CAM use in this sample was higher than that in previous studies, in which approximately half of the participating parents reported CAM use for their children with ASD [10,25]. The findings regarding dietary supplements and special diets were broadly consistent with previous reports [10,12]. Importantly, the questionnaire used the term “holding therapy” without a detailed operational description; therefore, the high reported frequency cannot be assumed to represent a single standardised intervention.

Parents perceived holding therapy and facilitated communication as the most effective interventions. However, the parent-reported perceived benefits observed in this survey should not be interpreted as evidence of clinical efficacy, and terminological ambiguity is an important limitation when interpreting these findings.

The main reasons for CAM use were parents’ desire to try every possible option for their child, reduce stress, and improve their child’s quality of life. Decisions were also influenced by positive experiences reported by other families and by a desire to reduce medication side effects. Some parents may perceive dietary supplements, diets, and vitamins as low-risk options even when their benefits are uncertain [10]. This highlights the need for clinicians to discuss both potential benefits and risks using evidence-based, non-judgmental communication [26]. Caution is required when interpreting facilitated communication (FC), which involves a person with a disability selecting letters, pictures, or objects while receiving physical support from a facilitator. Systematic reviews have not established independent authorship of FC-generated messages, and the American Speech-Language-Hearing Association considers FC a discredited technique that should not be used because of its lack of validity and documented risks [27,28,29]. FC should therefore be distinguished from evidence-based augmentative and alternative communication approaches that support independent communication.

Most participating parents were welleducated; however, fewer than one fifth reported their child’s physician as a source of information about CAM, whereas more than half relied on social media, consistent with previous research [26]. A study conducted in Jordan reported that physicians actively advised families about CAM methods and represented an important source of information [10].

Although more than two thirds of parents reported informing physicians about all or some of the CAM methods used, approximately one third stated that physicians had never asked about CAM use. These findings support the need to strengthen open family–clinician communication and for healthcare providers to create a respectful, non-judgmental environment in which CAM use can be discussed.

No consistent association between child sex and CAM use was observed across the two CAM categories. However, male sex was associated with greater use of mind and body practices in the corresponding regression model; this finding should be interpreted cautiously given the exploratory nature of the analysis [10,22].

Parental education level was not significantly associated with CAM use in this sample, which differs from findings reported in other studies [10,22,26]. This may partly reflect the overrepresentation of parents with higher levels of education in our sample. We also found no significant association between sufficient health literacy and CAM use compared with inadequate health literacy [30]. Although health literacy is related to education, it is a multidimensional construct that includes access to and processing of health information, healthcare use, disease prevention, and health promotion [30].

Better verbal ability was associated with greater use of natural products/biologically based therapies and mind and body practices, but the estimate was imprecise and should be interpreted cautiously. Other studies have reported no association between verbal ability and CAM use [10]. This association may reflect confounding by developmental severity, age, parental expectations, access to therapy, or other unmeasured characteristics associated with both verbal ability and the choice of interventions. Because this study was cross-sectional, temporal direction and causality be established.

It should be noted that some Arab countries have taken steps toward integrating CAM methods into conventional healthcare. Saudi Arabia is implementing healthcare reform as part of its Vision 2030 strategy, including a centre focused on CAM [31]. Moreover, the national health systems of Egypt, Jordan, Kuwait, Saudi Arabia, and the UAE incorporate herbal remedies as part of CAM alongside Western medical care [32].

### Strengths and Limitations

This study has several strengths. It examined CAM use among families of children with ASD attending five autism centres in five Arab countries using a common survey approach, providing information from settings that remain relatively underrepresented in the CAM literature. It also explored parents’ perceived effectiveness, motivations, information sources, disclosure to healthcare professionals, health literacy, and several parent- and child-related factors.

Several limitations should be considered. Convenience sampling and recruitment from only one autism centre in each country limit the generalisability of the findings. In addition, the analytical dataset did not retain a reliable centre/country identifier for individual respondents; therefore, the final distribution of participants across the five centres could not be reconstructed, and centre- or country-specific analyses were not possible. The findings should consequently be interpreted as arising from a centre-based multicountry sample rather than as nationally representative estimates. Selection bias is also possible because families receiving specialised autism services, particularly those with greater interest in CAM, may differ from the wider population.

The small sample size and sparse data for some CAM modalities reduced the precision of some multivariable estimates, as reflected by the wide confidence intervals. The regression findings should therefore be considered exploratory and interpreted cautiously. The study also relied on parent-reported information, which may be affected by recall, social desirability, and expectancy biases. Perceived effectiveness was not assessed using standardised clinical outcomes and should not be interpreted as evidence of therapeutic efficacy. Furthermore, the cross-sectional design does not establish causality, and grouping heterogeneous CAM modalities into broader categories may have obscured differences between individual interventions. Future larger, representative, multicentre, and longitudinal studies using standardised CAM definitions and objective outcome measures are warranted. Finally, the HLS-EU-Q16 response format was slightly modified by including a neutral category; therefore, the conventional health literacy categories used descriptively in this study may not be directly comparable with those obtained using the standard response format.

## 5. Conclusions

CAM use was common among families attending the five participating autism centres. Parents most often reported dietary supplements and the practice labelled “holding therapy”. The main reasons for CAM use were the desire to try all possible therapy options and to improve the child’s quality of life. However, some reported modalities have limited evidence of benefit, are not recommended for use in ASD, and may carry safety and ethical concerns. Healthcare professionals should proactively provide balanced, evidence-based information and create opportunities for non-judgmental discussion of CAM use, allowing families to make informed decisions. Improving health literacy and access to reliable information may support safer decision-making. As this convenience sample was drawn from one centre in each of the five included countries, the findings should not be generalised to national or regional populations. Future studies should use larger, more representative samples and may include comparison groups from the general population to distinguish ASD-specific patterns from broader cultural or household CAM practices.

## Figures and Tables

**Table 1:** Demographic characteristics of parents and children with ASD (*n = 142*).

Sociodemographic Data	*n* (%)
Age of parents	
Father	
23–29	5 (3.5%)
30–39	34 (23.9%)
40–49	69 (48.6%)
≥50	31 (21.8%)
Dead father	3 (2.1%)
Mother	
23–29	14 (9.9%)
30–39	65 (45.8%)
40–49	53 (37.3%)
≥50	10 (7.0%)
Place of residence	
City	120 (84.5)
Village	22 (15.5)
Level of education of parents	
Father	
Primary school	17 (12.0%)
Secondary school	38 (26.8%)
Higher education	87 (61.3%)
Mother	
Primary school	10 (7.0%)
Secondary school	37 (26.1%)
Higher education	95 (66.9%)
Monthly family income	
≤500 $	55 (38.7%)
501–1000 $	23 (16.2%)
1001–1500 $	16 (11.3%)
≥1500 $	18 (12.7%)
Prefer not to answer	30 (21.1%)
Number of siblings	
One child	19 (13.4%)
2–3	59 (41.5%)
>3	64 (45.0%)
Sex of child	
Male	117 (82.4%)
Female	25 (17.6%)
Age of children with ASD	
1–4 years	33 (23.2%)
5–9 years	49 (34.5%)
10–14 years	54 (38.0%)
>15 years	6 (4.2%)

**Table 2:** Clinical characteristics of children with ASD.

Characteristics	*n* (%) (*n = 142*)
Diagnosis	
Autistic disorder	125 (88.0%)
Pathological demand disorder	8 (5.6%)
Asperger syndrome	7 (4.9%)
Other	2 (1.4%)
Child verbal ability	
Does not talk	58 (40.8%)
Uses single words	23 (16.2%)
Uses two- or three-word phrases	27 (19.0%)
Uses sentence with four or more words or complex sentences	34 (24.0%)
Comorbidity	
ADHD (Attention deficit hyperactivity disorder)	110 (77.5%)
Feeding problems	51 (35.9%)
Sleep disturbances	44 (31.0%)
Epilepsy	10 (7.0%)

**Table 3:** CAM use and perceived effectiveness of CAM modalities among parents of children with ASD (*n = 142*).

	CAM Use		Effectiveness of CAM Use Among the Ever CAM Users **			
	Used in the Last 6 Months	Ever Used * n (%)	Not Effective *n* (%)	Neutral *n* (%)	Effective *n* (%)	No Response
Natural products/Biologically based therapies						
Multivitamin	4 (2.8)	75 (52.8)	26 (34.7)	32 (42.7)	9 (12.0)	8 (10.6)
Honey	2 (1.4)	48 (33.8)	22 (45.9)	12 (25.0)	6 (12.5)	8 (16.6)
Vitamin B6	0 (0)	43 (30.3)	18 (41.9)	17 (39.5)	5 (11.6)	3 (7.0)
Fish oil	18 (12.7)	60 (42.3)	30 (50.0)	13 (21.7)	12 (20.0)	5 (8.3)
Dates	10 (7.0)	43 (30.3)	23 (53.5)	14 (32.5)	3 (7.0)	3 (7.0)
Almond	9 (6.3)	39 (27.5)	23 (58.9)	12 (30.8)	1 (2.6)	3 (7.7)
Algae and herbs	7 (4.9)	33 (23.2)	23 (69.7)	4 (12.1)	3 (9.1)	3 (9.1)
Vitamin B12	12 (8.5)	36 (25.4)	18 (50.0)	11 (30.6)	4 (11.1)	3 (8.3)
Diets (total)	20 (14.1)	52 (36.6)				
Gluten free	6 (4.2)	42 (29.6)	23 (54.8)	7 (16.7)	9 (21.4)	3 (7.1)
Low sugar	8 (5.6)	41 (28.9)	24 (58.5)	8 (19.5)	7 (17.1)	2 (4.9)
Egg free	4 (2.8)	36 (25.4)	21 (58.3)	11 (30.6)	1 (2.8)	3 (8.3)
Soy free	3 (2.1)	33 (23.2)	19 (57.5)	6 (18.2)	5 (15.2)	3 (9.1)
Casein free	8 (5.6)	37 (26.1)	25 (69.4)	4 (11.1)	5 (14.9)	2 (5.6)
Aroma therapy	2 (1.4)	30 (21.1)	16 (53.4)	4 (13.3)	7 (23.3)	3 (10.0)
Chelation	8 (5.6)	34 (23.9)	22 (64.7)	3 (8.8)	8 (23.5)	1 (3.0)
Oxytocin therapy	8 (5.6)	24 (16.9)	20 (83.4)	2 (8.3)	2 (8.3)	0 (0)
Mind and body practices						
Holding therapy	15 (10.6)	73 (51.4)	21 (28.8)	15 (20.5)	33 (45.2)	4 (5.5)
Pet therapy	6 (4.2)	45 (31.7)	16 (35.6)	14 (31.1)	12 (26.7)	3 (6.6)
Massage	6 (4.2)	37 (26.1)	19 (51.4)	8 (21.6)	10 (27.0)	0 (0)
Facilitated communication	25 (17.6)	55 (38.7)	21 (38.2)	11 (20.0)	21 (38.2)	2 (3.6)
Hyperbaric oxygen therapy	4 (2.8)	33 (23.2)	23 (69.7)	4 (12.1)	4 (12.1)	2 (6.1)
Sensory integration therapy	12 (8.5)	41 (28.9)	22 (53.7)	7 (17.1)	12 (29.2)	0 (0)
Music therapy	8 (5.6)	37 (26.1)	20 (44.0)	8 (21.7)	7 (18.9)	2 (5.4)
Cupping	12 (8.5)	36 (25.4)	23 (53.9)	5 (13.9)	7 (19.4)	1 (2.8)
Acupuncture	4 (2.8)	27 (19.0)	23 (85.2)	2 (7.4)	2 (7.4)	0 (0)
Acupressure	5 (3.5)	25 (17.6)	21 (84.0)	3 (12.0)	1 (4.0)	0 (0)
Biofeedback	7 (4.9)	27 (19.0)	19 (70.4)	5 (18.5)	2 (7.4)	1 (3.7)
Packing therapy	7 (4.9)	29 (20.4)	14 (48.4)	5 (17.2)	7 (24.1)	3 (10.3)
Deep pressure	10 (7.0)	30 (21.1)	20 (66.6)	3 (10.0)	4 (13.4)	3 (10.0)
Osteopathy	8 (5.6)	27 (19.0)	19 (70.4)	4 (14.8)	3 (11.1)	1 (3.7)
Other						
Homeopathy	9 (6.3)	26 (18.3)	20 (77.0)	2 (7.7)	4 (15.3)	0 (0)

* The ever-used group includes all respondents who reported using a CAM method either during the last 6 months or at any previous time for their child with ASD. Recent use is therefore included within total ever use, and the two columns should not be summed. ** The perceived effectiveness of CAM use was calculated among the ever CAM users (the denominator is the ever-used group in each line).

**Table 4:** Binary logistic regression analysis of sociodemographic and medical variables and parental health literacy by main CAM group (*n = 142*).

CAM Group	Factor	Level	Adjusted Odds Ratio [95% Confidence Interval]	*p*-Value
Natural products/Biologically based therapies	Child gender	Male/Female	0.92 [0.34–2.47]	0.884
	Education mother	Higher education/Primary education and high school	1.14 [0.43–3.01]	0.806
	Education father	Higher education/Primary education and high school	0.744 [0.28–1.94]	0.571
	Medication use	Used/Never used	2.32 [0.99–5.49]	0.065
	Comorbidity	≥2/0 or 1	0.54 [0.24–1.21]	0.154
	Verbal ability	Uses single words or 2–3-word phrases/Do not talk	1.17 [0.53–2.60]	0.724
		Uses short/complex sentences/Do not talk	**5.35 [1.58–18.56]**	**0.010**
	Health literacy	Problematic/Inadequate	0.92 [0.37–2.28]	0.859
		Sufficient/Inadequate	0.77 [0.31–1.89]	0.583
Mind and body practices	Child gender	Male/Female	**3.28 [1.23–8.92]**	**0.029**
	Education mother	Higher education/Primary education and high school	2.07 [0.88–4.91]	0.127
	Education father	Higher education/Primary education and high school	0.70 [0.29–1.63]	0.441
	Medication use	Used/Never used	1.86 [0.89–3.94]	0.125
	Comorbidity	≥2/0 or 1	1.04 [0.50–2.17]	0.928
	Verbal ability	Uses single words or 2–3-word phrases/Do not talk	1.38 [0.64–3.01]	0.442
		Uses short/complex sentences/Do not talk	**4.30 [1.66–11.23]**	**0.005**
	Health literacy	Problematic/Inadequate	1.28 [0.55–2.99]	0.593
		Sufficient/Inadequate	1.8 [0.79–4.09]	0.193

Reference groups are underlined. Statistically significant differences are shown in bold.

## Data Availability

Data are available on request due to legal and ethical restrictions. The data contain information about minority populations.

## Supplementary Materials

The following supporting information can be downloaded at: https://www.mdpi.com/article/10.3390/healthcare14193351/s1. Supplementary Table S1. Operational definitions and study classification of CAM modalities included in the questionnaire. The sources used to define and classify the modalities in Supplementary Table S1 are also included in the main reference list [13,23,25,26,27,28,29,30,31,32].

## Abbreviations

The following abbreviations are used in this manuscript:

CAM	Complementary and alternative medicine
ASD	Autism Spectrum Disorder
