# Supplementary material for: Complementary and Alternative Medicine Use Among Families of Children with Autism Spectrum Disorder Attending Five Autism Centres in Arab Countries: Associations with Sociodemographic Factors and Health Literacy
Source: Healthcare (Basel). 2026 Oct 8;14(19):3351. doi: 10.3390/healthcare14193351 (PMC13664931; doi:10.3390/healthcare14193351)
Supplement: Supplementary file 1 [file healthcare-14-03351-s001.zip › healthcare-4517759-supplementary.pdf]

Supplementary Table S1. Operational Definitions and Study Classification of CAM Modalities

The categories below reproduce the operational grouping used in the study questionnaire/analysis. They should not be interpreted as a universal taxonomy. CAM classification systems have evolved, and some modalities may also be used within conventional rehabilitation or supportive care.

Study category	Modality	Operational definition	Definition/classification source
Natural products / biologically based therapies	Multivitamin	A preparation containing multiple vitamins, sometimes with minerals, used as a dietary supplement.	Study questionnaire; NCCIH dietary-supplement framework [13].
Natural products / biologically based therapies	Honey	Honey consumed as a food or traditional natural product with the intention of supporting health.	Study questionnaire; prior CAM-in-ASD survey framework.
Natural products / biologically based therapies	Vitamin B6	Supplementation with pyridoxine (vitamin B6).	Study questionnaire; NCCIH ASD evidence summary [15].
Natural products / biologically based therapies	Fish oil	Supplementation with fish-derived oils rich in omega-3 fatty acids, principally EPA and DHA.	Study questionnaire; NCCIH ASD evidence summary [15].
Natural products / biologically based therapies	Dates	Consumption of date fruit as a traditional food/natural product for perceived health benefit.	Study questionnaire; culturally used natural product.
Natural products / biologically based therapies	Almond	Consumption of almonds as a food/natural product for perceived health benefit.	Study questionnaire; food-based natural product.
Natural products / biologically based therapies	Algae and herbs	Use of algae-derived products and/or herbal preparations for perceived health benefit.	Study questionnaire; NCCIH natural-product framework [13].
Natural products / biologically based therapies	Vitamin B12	Supplementation with cobalamin (vitamin B12).	Study questionnaire; dietary-supplement framework [13].
Natural products / biologically based therapies	Gluten-free diet	An elimination diet that excludes foods containing gluten, a protein found primarily in wheat, barley, and rye.	Study questionnaire; NCCIH special-diet discussion [15].
Natural products / biologically based therapies	Low-sugar diet	A dietary approach intended to reduce intake of added/free sugars and high-sugar foods.	Study questionnaire.
Natural products / biologically based therapies	Egg-free diet	An elimination diet that excludes eggs and foods containing egg ingredients.	Study questionnaire.
Natural products / biologically based therapies	Soy-free diet	An elimination diet that excludes soy and foods containing soy-derived ingredients.	Study questionnaire.
Natural products / biologically based therapies	Casein-free diet	An elimination diet that removes casein, the principal protein in milk and dairy products; it is often combined with a gluten-free diet.	Definitions CAM document; NCCIH special-diet discussion [15].
Natural products / biologically based therapies	Aromatherapy	Use of plant-derived essential oils, most often by inhalation or diluted topical application, as a complementary health approach.	NCCIH [16].
Natural products / biologically based therapies	Chelation therapy	Administration of chelating agents that bind certain metals so that they can be excreted; it is a medical treatment for specific heavy-metal poisoning and is not an established ASD treatment.	CAM evidences document; NCCIH [15].
Natural products / biologically based therapies	Oxytocin therapy	Use of synthetic oxytocin; in ASD research it has been investigated experimentally for possible effects on social and emotional functioning.	Definitions CAM document; NCCIH ASD evidence summary [15].
Mind and body practices	Holding therapy	The survey used the label 'holding therapy' without a more detailed operational definition. In the critical literature, coercive holding/attachment therapy involves physical restraint or manipulation; this must not be conflated with supportive holding or deep-pressure sensory input.	CAM evidences document; Mercer 2013 [23].
Mind and body practices	Pet therapy	Structured therapeutic or supportive interaction involving animals (animal-assisted intervention/therapy).	Study questionnaire; broad complementary/supportive-care terminology.
Mind and body practices	Massage	Manual manipulation of soft tissues using techniques such as stroking, kneading, or pressure.	NCCIH mind/body and physical approaches [13].

Mind and body practices	Facilitated communication	A facilitator-dependent technique in which a person with a disability selects letters, pictures, or objects while receiving physical support from a facilitator. It is distinct from independent AAC.	Hemsley et al. 2018 [27]; Schlosser et al. 2014 [28]; ASHA 2018 [29].
Mind and body practices	Hyperbaric oxygen therapy (HBOT)	A medical treatment in which a person breathes 100% oxygen inside a pressurized chamber.	Definitions CAM document; Kot et al./European code of good practice [17].
Mind and body practices	Sensory integration therapy	A clinical intervention using structured sensory-motor activities to support adaptive responses to sensory information; Ayres Sensory Integration® is a specific manualized approach with defined principles.	Definitions CAM document; study questionnaire. Classification as CAM is context-dependent.
Mind and body practices	Music therapy	Therapeutic use of music and musical interaction by a trained professional to address individualized goals.	NCCIH ASD evidence summary [15].
Mind and body practices	Cupping	A practice in which cups are applied to the skin to create suction; wet cupping additionally involves superficial skin incisions.	Study questionnaire; traditional/complementary practice.
Mind and body practices	Acupuncture	A traditional practice involving stimulation of specific body points, most commonly by insertion of thin needles through the skin.	NCCIH mind/body and physical approaches [13,15].
Mind and body practices	Acupressure	Manual pressure applied to points used in acupuncture traditions, without needle insertion.	Study questionnaire; traditional/complementary practice.
Mind and body practices	Biofeedback	A mind-body technique using monitoring instruments to provide real-time information about physiological processes so that individuals can learn to modify them.	Definitions CAM document; NCCIH [13].
Mind and body practices	Packing therapy	A practice described in the literature in which a child is wrapped in cold, wet sheets or towels and then covered with dry blankets to induce rewarming. It has been the subject of major ethical and safety concerns.	CAM evidences document; Amaral et al. 2011 [14].
Mind and body practices	Deep pressure	Firm tactile/proprioceptive sensory input delivered through pressure, compression, hugging, swaddling, stroking, or weighted/compressive materials. It is distinct from coercive holding therapy.	Definitions CAM document.
Mind and body practices	Osteopathy	A healthcare approach that may include hands-on assessment and manual techniques directed at the musculoskeletal system; scope and regulation vary by country.	Study questionnaire; complementary/manual-therapy terminology.
Other	Homeopathy	A system based on highly diluted preparations selected according to the homeopathic principle of 'like cures like'.	NCCIH [18].
